# The future of psychiatry

**DOI:** 10.17816/CP63

**Published:** 2021-03-20

**Authors:** Norman Sartorius

**Affiliations:** Association for the Improvement of Mental Health Programmes (AMH)

**Keywords:** future psychiatry, psychiatric education, prevention of mental illness, promotion of mental health, mental health, paradigms of psychiatry, будущее психиатрии, психиатрическое образование, профилактика психических заболеваний, укрепление психического здоровья, психическое здоровье, парадигмы психиатрии

## Abstract

Since the Second World War mental health programmes and psychiatry have made significant advances. Countries, as well as the United Nations, have recognized the magnitude and severity of mental health problems, and numerous national programmes have been launched to deal with them. Technology relating to the treatment of mental disorders has advanced and significant progress has been made in terms of knowledge regarding the functioning of the brain. The awareness of the need to protect the human rights of those with mental illness has increased. National and regional programmes against stigma and the consequent discrimination of those with mental illness, have been launched in many countries. Associations bringing together those who have experienced mental illness and their relatives, have come into existence in many countries.

While these are great steps forward, more work is necessary to complete these advances. In low- and middle-income countries, the vast majority of people with mental disorders do not receive adequate treatment. Even in highly industrialized countries, a third of people with severe forms of mental illness are not receiving the appropriate therapy. Laws concerning mental health are outdated in many countries. The protection of the human rights of the mentally ill is incomplete and imperfect. The emphasis on economic gain and the digitalization of medicine in recent years has not helped. On occasions, this has even slowed down the development of mental health services, and the provision of mental healthcare. Thus, psychiatry must still deal with the challenges of the past century, while facing new demands and tasks.

Among the new tasks for psychiatry are undoubtedly reforms which will allow (i) the provision of appropriate care of people with comorbid mental and physical disorders, (ii) the application of interventions leading to the primary prevention of mental and neurological disorders, and (iii) a radical reform of the education of psychiatrists and other mental health workers, dealing with mental illness. Collaboration with other stakeholders in the field of mental health and medicine, will be of crucial importance in relation to all these tasks.

It is possible that the future of psychiatry will be bright, not least because psychiatry has won several battles that it has fought since the middle of the last century. The first and possibly the most important of these battles, was the recognition – by the medical community, society and governments – that mental health matters, and that it is necessary to invest time and money into reducing the burden that mental disorders place on society. and help those who are suffering from mental disorders (as well as those who care for them). A number of countries have adopted national mental health programmes, and the United Nations has included the fight for better mental health among its sustainable development goals [1].

Another battle, fought over an even longer period, was the development and application of methods of treatment and rehabilitation, leading to the recovery and inclusion of those who have suffered from a mental illness by their ccommunity. In this respect also major advances have been made, and it is fair to say that the success of the treatment of mental disorders, is equal or better than the success of the treatment of other noncommunicable diseases.

The importance of reducing the stigma of mental illness – stigma being a major obstacle to all efforts in the field of mental health - has also been recognized, and anti-stigma programmes have been introduced in several European countries – including Denmark, the Netherlands and the United Kingdom – and elsewhere, e.g., in Australia, New Zealand and Canada [2].

The decades since the Second World War have also seen a significant increase in the number of psychiatrists – from around 50,000 in the 1950s to more than 250,000 today [3]. The numbers of other health workers, dealing with mental disorders, have also grown exponentially.

The list of successes and battles won does not end there: science has made major steps forward in its effort to understand the functioning of the brain. The fact that the provision of adequate mental healthcare, in partnership with general healthcare services and by general healthcare workers, can be provided even in very poor countries, has been demonstrated in carefully designed studies [4,5]. The human rights of those with mental illness have been recognized in numerous international and national statements and binding documents [6]. Organizations bringing together people who experienced mental illness, their family members and others willing to help people with mental illness lead a life worth living, in their community, have come into existence in many countries.

Although we can be proud of these successes, it is also clear that the battles mentioned above will continue. While the importance of helping people with mental illness and their families has been recognized in many countries, the budgetary allocations for mental health programmes remain low. Many of the countries which have agreed to pursue sustainable development goals, suffer from financial and other difficulties, and their efforts to achieve the goals which they agreed to pursue, have been feeble. The human rights of people with mental disorders are not sufficiently respected or protected, and the laws relevant to mental health care, are outdated in many countries. The gains made in terms of improved technology, in relation to the treatment of mental disorders, have not yet yielded the expected benefits: in low- and middle-income countries, up to 80% of all individuals with severe forms of mental illness, do not receive adequate treatment. Even in the industrially-advanced, rich countries, nearly one third of people with mental diseases, do not receive appropriate treatment [7]. The programmes launched to reduce stigma are a significant step forward, but they are programmes which are separate from the mental healthcare system: it is to be hoped that the fight against stigma will become an essential part of mental health service activities, and that this will be reflected in the budget of all psychiatric services and institutions. The associations, bringing together people with mental illnesses and their family members, often lack financial and administrative support and depend on a small number of activists, who vary in their capacity to be fully engaged which renders these organizations vulnerable and affects their ability to fulfil their goals.

It is also important to recognize that these developments are taking place in a world currently influenced by several major trends, directly relevant to the development of healthcare, in general and psychiatry, in particular. The first of these is “commodification” - the tendency to consider all activities and all developments in economic terms, as if they were forms of handling commodities, such as sugar, cotton, coal or timber [8]. Thus, a hospital will be considered a success if it can work in a manner that will result in financial gain, a profit; the treatment of an individual with an illness, will be considered a success, if it enables him or her to continue working and producing. Conversely the treatment would be a failure if it does not achieve this, regardless of the effects that it might have had in terms of quality of life or extension of life expectancy. Commodification results in changes in healthcare priorities and in the manner of providing services, and can be particularly detrimental to work in the fields of psychiatry or geriatrics.

Another major development in terms of importance for medicine and mental healthcare, is the digitalization of medicine and other social and industrial pursuits. While offering vast improvements in medical technology and diagnostics, and opening the door to telemedicine and telepsychiatry, digitalization also impacts the relationship between health workers and those who seek help and can dehumanize medicine. While an excellent tool, and potentially a good servant of medicine, digitalization has in many instances been allowed to become its master imposing ways of working and communicating that deny or eliminate empathy, reduce the sense of belonging and weaken humane interaction in the fabric of society.

While the digitalization of medicine and commodification may bring benefits to the practice of psychiatry but also harm it, there is another trend which might play an increasingly important role in the provision of mental healthcare: this is the rediscovery of the potential of self-help techniques and mutual help arrangements. The peer support system has been shown to facilitate and improve the care of patients with diabetes, as well as those with substance abuse problems. The mental health first-aid materials, produced in several countries, as well as the increasing emphasis on education of the population regarding health-promoting lifestyles, are indications of the recognition of the importance of self-help in medicine, in general and in psychiatry, in particular. It is to be hoped that the trend of facilitating self-help and mutual help will continue to grow, and that it will find support in general and in health education programmes.

If psychiatry is to be useful to society and to people with mental health problems in the future, it will have to expand its field of activity and add new pursuits to those of providing treatment for people with mental illness. It will have to seek a productive alliance with other disciplines of medicine, and with other professions, not only to improve the provision of care to people with comorbid mental and physical illness but also to benefit from advances of medicine in general.

Mental health programs will also have to make significant efforts to elevate the position of mental health on the scale of values of both individuals and societies. Once mental health is high on the scale of values held by individuals and society, and people consider it important mental health programmes will receive the moral and material support necessary to build and maintain comprehensive mental health programmes. The effort to promote mental health on the scale of values, as well as other tasks in the field of mental health, will have to be approached in collaboration with other stakeholders in the field of mental health, including, in particular, those who have experienced mental illness and their carers.

Another area of emphasis in which the psychiatrists of the future will need to invest time and effort, will be the significant expansion of programmes, focusing on the primary prevention of mental and neurological disorders. At present this area of work is neglected by psychiatrists, partly because the action necessary to introduce primary prevention is in the hands of other professions and institutions, and partly because psychiatrists are overburdened by the tasks related to the provision of care to people with mental illness. An example of primary prevention of a serious mental disorder, is the provision of iodine for women of childbearing age, which would prevent cretinism in their children; another example is involvement in perinatal care, to provide women with information about the upbringing of their children or the danger of foetal alcohol syndromes. Numerous other examples of action that could lead to the primary prevention of mental and neurological disorders could be listed: most, if not all of them, are in areas controlled by other professions or institutions. To influence these, psychiatrists will have to make a serious effort beyond their usual area of work.

Investing time and effort into introducing the primary prevention of mental and neurological disorders, is not the only area in which psychiatry will have to step outside its current principal areas of activity: another is the need to organize services for people, suffering from a mental as well as a physical disorder. Comorbidity is already prevalent and reduces the life expectancy of those with mental disorders by as much as 12 to 15 years: it is likely that the prevalence of comorbidity and multimorbidity will increase for a variety of reasons, including the extension of life expectancy and the improved survival rates of people, suffering from non-communicable diseases [9].

The current education of psychiatrists does not include systematic training in skills, likely to helpful in the development of mental health programmes as well as in the efforts to elevate mental health on the scale of values of individuals and societies. Skills of communication, such as those of negotiation and of public speaking, are tools which are useful in the process of convincing the many who can be usefully involved in the building of mental health programmes: they should be added to the postgraduate training of psychiatrists, which should also include appropriate instruction in public health, in legal matters and behavioural sciences. The organizers of training for psychiatrists must also consider ways of involving other stakeholders in the education of mental health workers. Among them should be family members and others who take care of severely mentally ill people in the community, social workers, nurses and other staff involved in the care of people with mental illness. Educators should also consider the best place to provide training for psychiatrists: learning about ways of reducing anxiety before an operation, should be provided in the surgical department, and the management of depression among those with diabetes should take place in the diabetic clinic, rather than in the department of psychiatry.

Training can provide skills and knowledge, and it can change attitudes: however, it cannot change the personality of the student. Educators should keep this in mind when selecting applicants for postgraduate training, and when advising graduates where they should seek work.

Psychiatrists must face these and other tasks which the future is placing before them. In addition, however, those engaged in mental health programmes should remember that they can and should contribute to making and keeping medical practice a humanitarian enterprise and that they should be a model of solidarity with people who are suffering severe illnesses and with those who provide care for them.
